# On the Valorization of *Arbutus unedo* L. Pomace: Polyphenol Extraction and Development of Novel Functional Cookies

**DOI:** 10.3390/foods12193707

**Published:** 2023-10-09

**Authors:** Hugo Duarte, Ceferino Carrera, María José Aliaño-González, Rocío Gutiérrez-Escobar, María Jesús Jiménez-Hierro, Miguel Palma, Ludovina Galego, Anabela Romano, Bruno Medronho

**Affiliations:** 1 MED—Mediterranean Institute for Agriculture, Environment and Development, CHANGE—Global Change and Sustainability Institute, Faculdade de Ciências e Tecnologia, Universidade do Algarve, Campus de Gambelas, Ed. 8, 8005-139 Faro, Portugal; hmduarte@ualg.pt (H.D.); lgalego@ualg.pt (L.G.); aromano@ualg.pt (A.R.); bfmedronho@ualg.pt (B.M.); 2 Departamento de Química Analítica, Facultad de Ciencias, Universidad de Cádiz, 11510 Cadiz, Spainmiguel.palma@uca.es (M.P.); 3 IFAPA Rancho de la Merced, Ministry of Agriculture, Fisheries, Water and Rural Development, Junta de Andalucía, Cañada de la Loba, 11471 Jerez de la Frontera, Cádiz, Spain; rocio.gutierrez@juntadeandalucia.es (R.G.-E.); mariaj.jimenez.hierro@juntadeandalucia.es (M.J.J.-H.); 4 Instituto Superior de Engenharia, Universidade do Algarve, Campus da Penha, 8005-139 Faro, Portugal; 5 FSCN—Fibre Science and Communication Network Research Center, Surface and Colloid Engineering Deparment, Mid Sweden University, SE-851 70 Sundsvall, Sweden

**Keywords:** *Arbutus unedo* L., distillate pomace, ultrasound-assisted extraction, antioxidant activity, functional cookies

## Abstract

The fruits of *Arbutus unedo* L. have a crimson colour and are enriched with remarkable concentrations of bioactive compounds such as anthocyanins and polyphenols. These fruits are commonly used in the production of a Portuguese Protected Geographical Indication distillate called “Aguardente de Medronho”. During this process, a solid pomace is generated and presently discarded without valuable applications. In this work, two strategies have been developed for the valorisation of *A. unedo* pomace. The first approach considers the extraction of polyphenols from this by-product through the optimization of an ultrasound-assisted method using a Box-Behnken design coupled with response surface methodology. The results indicate that the temperature and the percentage of methanol, along with their interaction, significantly influence the total concentration of polyphenols and the antioxidant activity of the extracts obtained. The optimal conditions identified consider the extraction of 0.5 g of sample with 20 mL of a solvent containing 74% MeOH (aq), at a pH of 4.8, maintained at 70 °C for 15 min. On the other hand, the second valorisation strategy considered the use of *A. unedo* pomace in the development of functional cookies. The incorporation of 15–20% pomace in the cookie formulation was well-received by consumers. This incorporation results in an intake of ca. 6.55 mg of polyphenols per gram of cookie consumed, accompanied by an antioxidant activity of 4.54 mg Trolox equivalents per gram of cookie consumed. Overall, these results encourage the employment of *A. unedo* pomace either as a reliable source of extracts enriched in polyphenols or as a nutraceutical active ingredient in functional cookies, thereby positively impacting human health.

## 1. Introduction

*Arbutus unedo* L. is a shrub/small tree belonging to the *Ericaceae* family that can reach a height of ca. 1.5 to 3.0 m [1]. This species is endemic to the Mediterranean flora and is predominantly found in southern Europe, northern Africa, certain regions of the United Kingdom, and the Macaronesian islands. The *A. unedo* holds significant importance in reforestation programs implemented in countries, such as Greece, Italy, Portugal, and Spain, mainly due to its rapid regeneration capacity following wildfires [2], which are common in these regions during dry seasons.

The fruits of the *A. unedo* measure approximately 2.0–3.0 cm in diameter, and are known for their pleasant and sweet taste when fully ripe. However, these fruits have a high sugar content and a delicate texture, rendering them highly perishable [3]. As a result, fresh consumption is still uncommon, thus being usually processed into jams, marmalades, or beverages. In Portugal, the fruits are traditionally used to produce a Portuguese Protected Geographical Indication distillate known as “Aguardente de medronho”. Apart from sugars, the fruits of *A. unedo* have revealed also significant concentrations of fatty acids, vitamin C, fibres, and polyphenols [4,5].

Extensive biological and health-related properties have been associated to the presence of polyphenols in *A. unedo* fruits, such as antibacterial, antifungal, antiparasitic, antiaggregant, antidiabetic, antihypertensive, antitumoral, antioxidant, and anti-inflammatory effects [6,7,8]. Notably, these fruits have demonstrated promising advancements in the treatment of renal, gastrointestinal, dermatological, urological, cardiovascular, and hypertensive diseases [9,10,11,12,13]. However, studies regarding the characterization and valorisation of the residues generated from the *A. unedo* distillation are still scarce. Alexandre et al. [14] identified the presence of phenolic compounds, such as pyrogallol, gallic acid, catechol, and protocatechuic acid, within the pomace. Interestingly, Rodrigues et al. [15] have shown that the fermentation and/or distillation significantly increase the total flavonoid content in the pomace, being ca. 2 times higher than in the fresh *A. unedo* fruits.

Undoubtedly, the distillate residues hold a very appealing chemical profile with high potential for valorisation in medicine, pharmaceutical and nutraceutical industries, and even the agri-food industry itself [5,6,8]. A crude estimation from local authorities suggests that in Portugal are produced ca. 4500–6000 tons of pomace per year. Therefore, the first strategy considered in this research for pomace valorisation relies on the extraction of its polyphenols. As mentioned, these secondary metabolites possess remarkable properties that make them interesting for their application in several fields. Among the different approaches, ultrasound-assisted extraction (UAE) is an analytical technique that has experienced a noteworthy rise in its utilization, mainly within the food industry in recent years. This upsurge can be attributed to its numerous advantageous features, which classify it as an environmentally friendly extraction method. In particular, UAE offers rapid analysis, reduced energy and solvent consumptions due to the cavitation phenomenon [16]. Cavitation-induced cell disruption facilitates enhanced interaction between solid matrices and solvents, thereby increasing the purity and yield of extractions, all achieved under milder conditions, reducing significantly the environmental impact [17]. Furthermore, UAE eliminates the need for extreme temperatures, thereby preventing the degradation of bioactive compounds, such as polyphenols. UAE has been extensively utilized in the extraction of phenolic compounds from various food sources, encompassing both primary products and by-products [18,19]. Notable examples include mushrooms [20], blackcurrants [21], potatoes [22], moringa leaves [23], onion bulbs [24], maritime pine residues [25], and even fresh *A. unedo* fruits [26]. Nevertheless, to the best of our knowledge, UAE has never been used in *A. unedo* pomaces. Therefore, the first goal of this study focus on the optimization of an UAE-based method to produce polyphenols-enriched extracts from pomace residues derived from *A. unedo* fruits distillation. The second goal of this study considers the direct application of *A. unedo* pomace in the development of novel functional cookies, with superior antioxidant capacity. Overall, this research expects to propose two novel strategies that contribute to the valorisation of a local residue currently discarded but with high appealing health-related properties, rendering potential application in various fields, such as nutraceuticals, medicine, cosmetics, pharmacology, and the food industry.

## 2. Materials and Methods

### 2.1. Arbutus unedo L. Pomace Processing

The *A. unedo* pomace was obtained from a small distillery (Monchique, Faro, Portugal). Briefly, the distillation process was performed as follows. After harvesting, the fresh fruits were sorted (removing peduncles, leaves, and any unripe fruits), and placed in a plastic barrel to which a certain amount of water was added (typically, up to 3 L per 15 kg of fruits). The barrels where fermentation took place were sealed to minimize unwanted oxidation. Note that the alcoholic fermentation of *A. unedo* fruit takes place spontaneously with naturally occurring yeasts. At the end of the fermentation (up to 2 months), the fermented fruits and liquid were transferred to a traditional copper-based distilling system (maximum capacity of 120 L) and the distillation process started. The alcohol content was carefully controlled to achieve the desired percentage. At the end of the process, the *A. unedo* pomace is usually discarded, and the copper-distillation apparatus cleaned for subsequent runs.

The collected pomace was frozen at a temperature of −20 °C and subsequently lyophilized to remove all traces of water. The freeze-drying process was conducted using a VirTis BenchTop Pro Freeze Dryer (SP Industries, Warminster, PA, USA). After lyophilization, the samples were further ground using an electric MKM6003 coffee grinder (BSH Electrodomésticos España S.A., Spain). The resulting powder was stored at −20 °C until further use.

### 2.2. Chemicals

Methanol (MeOH) of analytical grade was acquired from Thermo Fisher Scientific (Waltham, MA, USA). Formic acid and hydroxide sodium, analytical grade, were purchased from Merck (Darmstadt, Germany) and used for the pH regulation. Gallic acid with a purity of 95% (Sigma-Aldrich Chemical Co., St. Louis, MO, USA) was used as the standard for the quantification of total polyphenols. The antioxidant activity was accessed using following the standards: 2,2-diphenyl-1-picrylhydrazyl (DPPH) from Sigma-Aldrich, and 6-hydroxy-2,5,7,8-tetramethylchroman-2-carboxylic acid (Trolox) from Thermo Fisher Scientific. Distilled-grade water was obtained from a purification system installed at the University of Algarve.

### 2.3. Polyphenols Extraction

#### 2.3.1. Ultrasound-Assisted Extraction System

An ultrasonic bath S 100 H (220–240 V, 550 W) (Elma Hans Schmidbauer GmbH & Co. KG, Singen, Germany), equipped with a 9 L water bath and temperature control, was used for the USE method. For each extraction, 0.5 g of *A. unedo* pomace powder was weighed and placed in an Erlenmeyer flask. The appropriate volume of solvent, taking into account the percentage of methanol (%MeOH) and pH requirements, was added to the flask. Subsequently, the samples were placed in the temperature-controlled water bath and maintained at a fixed temperature for 15 min, with the ultrasonic frequency set at 50 Hz and power of 550 W. These parameters have been selected because they have been shown to be influential variables in the extraction of polyphenols with UAE in similar matrices in investigations previously carried out by our research group [21,25,27]. Following the extraction process, the obtained extracts were filtered using a Buchner funnel and a vacuum pump. The filtered extracts were then collected in 25 mL volumetric flasks, which were filled up to the mark with the corresponding solvent. The extracts were then stored at 5 °C until further analysis. All extractions were performed in duplicate.

#### 2.3.2. Ultrasound-Assisted Extraction System

In order to evaluate the impact of different variables on the concentration of polyphenols in the extracts and to determine the optimal conditions for maximizing their content, an experimental design was employed. A Box-Behnken design coupled with response surface methodology (BBD-RSM) was chosen.

The BBD-RSM uses a factorial design where three levels, (−1) as the lowest level, (0) as the intermediate level, and (1) as the highest level, are assigned to each factor. This design forms a cube-shaped experimental space that includes a central point and midpoints at the edges, effectively excluding axial points [28]. It is important to note that the BBD-RSM reduces the maximum number of experiments required while obtaining the same information of other three-level full factorial designs, thus making it a more efficient approach [29]. Moreover, the design of experiments used in this study avoids extreme conditions that could result in economic costs, power consumption, or result in polyphenol degradation. By employing this approach, the experiments conducted strike a balance between obtaining meaningful results and ensuring practical feasibility.

Four key variables were selected for evaluation, namely: the solid-to-solvent ratio (0.5 g/10–15–20 mL of solvent), the %MeOH in the solvent (25–50–75%), the pH of the solvent (2–4–7), and the extraction temperature (30–50–70 °C). The range of these variables was selected based on existing literature on similar matrixes [20,21,26]. These supposed a total of 27 experiments (Table 1) that were performed in duplicate.

Two response variables were considered for optimization: (i) the concentration of total polyphenols and (ii) the antioxidant activity. The methodology used to determine these parameters is described next. The BBD-RSM was employed to establish the relationship between the response variables and the independent variables following the Equation (1):

Y = *β*_0_ + *β*_1_X_1_ + *β*_2_X_2_ + *β*_3_X_3_ + *β*_4_X_4_ + *β*_12_X_1_X_2_ + *β*_13_X_1_X_3_ + *β*_14_X_1_X_4_ + *β*_23_X_2_X_3_ + *β*_24_X_2_X_4_ + *β*_34_X_3_X_4_ + *β*_11_X_1_^2^ + *β*_22_X_2_^2^ + *β*_33_X_3_^2^ + *β*_44_X_4_^2^ (1)

In this equation, Y represents the response variable (total content of polyphenols or antioxidant activity of the extracts), while *β*_0_ corresponds to the intercept term. The independent variables are denoted as X_1_ (solid-to-solvent ratio), X_2_ (%MeOH), X_3_ (pH), and X_4_ (temperature). The linear coefficients are represented by *β_i_*, the cross-product coefficients by *β_ij_*, and the quadratic coefficients by *β_ii_*.

#### 2.3.3. Total Content of Polyphenols

The optimization of the total polyphenol content was a crucial objective in this study to ensure the maximum extraction of *A. unedo* pomace. The evaluation of this variable was conducted using the total polyphenol index, which was accessed by measuring the absorbance of the extracts at 280 nm, utilizing a Genesys 10 uv spectrophotometer (Thermo Scientific, Waltham, MA, USA). A calibration curve was prepared (i.e., from 0 to 800 mg/L) using gallic acid as the standard. The absorbance values obtained were correlated with the corresponding gallic acid concentrations. The resulting calibration curve equation was Y = 6.08X − 0.01, where Y represents the absorbance and X represents the gallic acid concentration in mg/L. The regression coefficient (R^2^) for the calibration curve was determined to be 0.9994. The total polyphenol concentration in the extracts was expressed as mg/g dry weight (dw).

#### 2.3.4. Antioxidant Activity of Pomace Extracts

The optimization of the extraction process also aimed at maximizing the antioxidant capacity of the extracts, as it ensures the presence of not only high levels of polyphenols but also their associated beneficial health-related properties.

The DPPH (2,2-diphenyl-1-picrylhydrazyl) method was employed [30] to evaluate the antioxidant activity of the extracts. The typical procedure involves mixing 300 µL of a DPPH solution (90 µM DPPH in MeOH:H_2_O, 80:20% *v*/*v*) with 30 µL of the pomace extract and 570 µL of a MeOH:H_2_O solution (80:20% *v*/*v*). The mixture was kept at room temperature in the dark for 30 min before measuring the absorbance at 515 nm. Trolox, a water-soluble vitamin E analogue, was selected as the standard antioxidant compound. A calibration curve was constructed using Trolox concentrations ranging from 0 mM to 0.30 mM, generating a six-point regression curve. The resulting calibration curve was Y = 221.57X + 2.51, with an obtained R^2^ of 0.9915. The antioxidant activity of the extracts was expressed as milligrams of Trolox equivalents (TE) per gram of dry weight (mg TE/g dw).

### 2.4. Identification of Polyphenols by UHPLC-PDA-QToF-MS-QDa

The extracts obtained under optimal conditions were analyzed using a previously developed method by our research group [31]. Prior to the analysis, the extracts were filtered through a 0.20 µm nylon syringe filter from Membrane Solutions (Dallas, Texas, USA), and 3 µL of the filtered extract was injected. Ultra-high-Performance Liquid Chromatography coupled with a Photodiode Array (PDA) detector system and a Quadrupole-Time-of-Flight Mass Spectrometric system with a QDa detector (UHPLC-PDA-Q-ToF-MS-QDa) were used for the identification of polyphenols. The UHPLC-PDA-Q-ToF-MS-QDa system employed was the Xevo G2 QToF from Waters Corp. (Milford, MA, USA) with an C18 analytical column with a particle size of 1.7 µm and dimensions of 2.1 mm × 100 mm (AQUITY UPLC CSH C18, Waters Corp.). The analysis was performed using a binary solvent system was used, consisting of phase A (2% solution of formic acid in water) and phase B (2% formic acid solution in methanol). The flow rate during the analysis was maintained at 0.4 mL/min, and the wavelengths were fixed at 280, 320 and 560 nm.

The main compounds identified according to their retention time and molecular weight were the following: gallic acid (1.000 min, *m*/*z* 170); 5-*O*-galloylquinic acid (1.870 min, *m*/*z* 344); and ethyl gallate (5.935 min, *m*/*z* 198). The area of these compounds was calculated in the extracts obtained under optimal conditions. Later, the extracts obtained from the cookies with different percentages of *A. unedo* pomace were also analyzed to evaluate the influence of backing process.

### 2.5. Development of Functional Cookies from Arbutus unedo L. Pomace

Numerous research studies have demonstrated the significant health-related properties of polyphenols, including their antioxidant, antimicrobial, and antifungal effects [7,25,32]. In addition, polyphenols have been previously identified in the *A. unedo* distillate residues [14,15] which opens the possibility of valorising this pomace. In the present study, the optimization of a method for obtaining polyphenols-enriched extracts from *A. unedo* pomace using USE was performed (see Section 2.3.1 for details). Additionally, the direct use of the pomace for the development of novel functional cookies was evaluated. In this regard, a standard recipe was performed with the following ingredients: 250 g of butter, 150 g of sugar, 1 large egg, 550 g of baking flour, and 5 mL of vanilla essence. Each batch resulted in 40 units. The process involved beating the butter and sugar together, adding the vanilla essence and the egg while continuing to beat until well-integrated. The flour (with or without *A. unedo* pomace powder) was then added, mixed using a blender followed by manual mixing. The dough was rolled out to a thickness of approximately ½ cm on a sheet of paper, refrigerated for 30 min, cut into desired shapes using a cookie cutter, and refrigerated again for additional 30 min. The cookies were baked at 180 °C with top and bottom heat without convection for 10–12 min, and then allowed to cool at room temperature.

As mentioned above, the baking flour was partially replaced (i.e., 0%, 10%, 15%, 20%, 40%, and 60%) with *A. unedo* pomace powder. Pomace powder above 60% was not evaluated since the consistency of the dough was compromised.

The sensory evaluation of the cookies was conducted through a Google Form survey. The survey assessed the panelists’ perceptions of the cookies based on several attributes, including visual appearance, aroma, texture, and taste. The evaluation involved a total of 82 panelists from Spain (University of Cádiz, Campus de Puerto Real, Cádiz) and IFAPA (Rancho La Merced, Jerez de la Fra.) and 20 panelists from Portugal (University of Algarve, Gambelas and Penha Campi, Faro), utilizing specially designated testing rooms for this purpose. Each panelist had a QR code available in their cubicle which give access to the survey. The survey QR code and photos from the testing rooms are shown in Figure S1. It is important to note that each sensory analysis panelist voluntarily agreed to participate in the sensory analysis, that they were previously informed of the test they were going to perform, as well as the ingredients they were going to consume in order to avoid possible allergies (information that is also included in the survey), and about the purpose of the study in order to ensure compliance with the ethical bases of any study.

The total polyphenol index and antioxidant activity of the cookies were evaluated as described in Section 2.3.3 and Section 2.3.4, respectively. To accomplish this, the cookies were grounded using a coffee grinder, and the optimal extraction conditions obtained with the BBD-RSM methodology were applied to obtain the polyphenol-enriched extracts from the cookies.

### 2.6. Data Analysis and Statistics

The BBD-RSM design was performed using the Statgraphic Centurion software (version XVII) from Statgraphics Technologies, Inc. (The Plains, VA, USA). The data obtained were compared and grouped based on the least significant difference (LSD) method, response surface regression techniques, analysis of variance (ANOVA) and the Fisher test. The significance level was set at 95%, corresponding to a *p*-value ≤ 0.05.

For the surveys regarding the *A. unedo* pomace-based cookies, Google Forms were utilized to collect the data. The obtained data were subjected to statistical analysis using Python programming within Jupyter Notebook (Anaconda, version 2.3.2).

## 3. Results and Discussion

### 3.1. Box-Behnken Design

First, the effect of four selected extraction variables was evaluated; the solid-to-solvent ratio, %MeOH, pH of the solvent, and the extraction temperature. A BBD-RSM design was used, resulting in a total of 27 experiments, randomly performed in duplicate. Samples were processed as described in experimental section. Note that the amount of sample per assay (0.5 g), the frequency (50 Hz), the power (550 W), and the extraction time (15 min) were kept constant based on the prior expertise of the research group.

The extracts were treated as previously described and the concentration of total polyphenols was determined and expressed as milligrams per gram of dry sample. The resulting data were then compared to the predicted values derived from the BBD-RSM design (Table 1). The average difference observed was 8.10% (ranging from 1.24% to 15.87%). The developed method demonstrated an R^2^ value of 0.71, indicating a reasonable fit, while the Durbin-Watson *p*-value was calculated as 1.77, falling below the significance threshold of 0.05. This indicates that no significant differences were found between the predicted and observed values.

**Figure 1:**
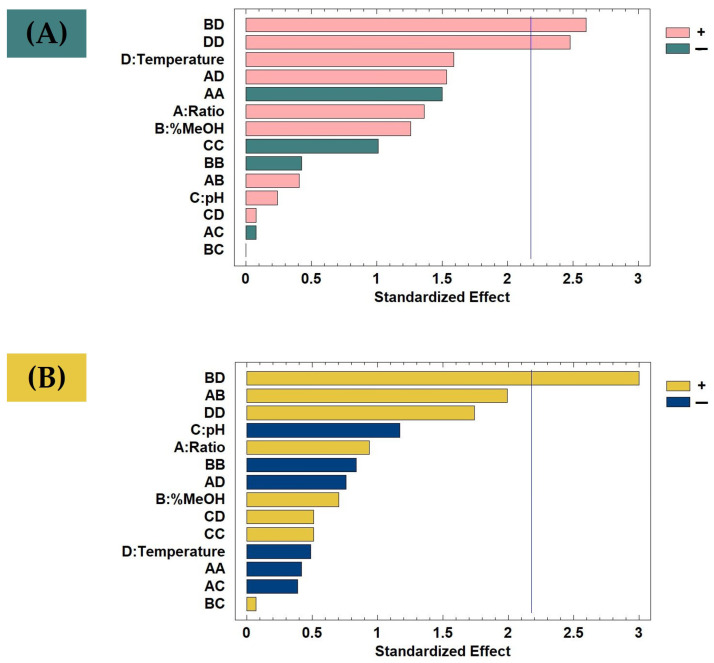
Pareto Chart of BBD-RSM analysis of (**A**) total polyphenols extracted from *A. unedo* pomace and (**B**) antioxidant activity of the extracts. The vertical line marks the 95% confidence level.

After confirming the normal distribution of the results, an ANOVA was performed. The results of this analysis were presented in a Pareto chart (Figure 1A). The analysis showed that the quadratic interaction of temperature (*p*-value: 0.0290) and the interaction between %MeOH and temperature (*p*-value: 0.0232) had significant effects on the extraction of total polyphenols from *A. unedo* pomace. Both variables demonstrated a positive effect, indicating that higher values, within their respective ranges, correlated with increased concentrations of total polyphenols. Consequently, the optimal extraction conditions were set as follows: 0.5 g of sample diluted in 20 mL of solvent (composed of 74% MeOH and having a pH of 4.8), with an extraction time of 15 min at 70 °C.

The obtained second order Equation (2) for the total polyphenol concentration was:

Y = 102.905 + 2.886·X_1_ − 0.785·X_2_ + 4.717·X_3_ − 3.509·X_4_ − 0.184·X_1_^2^ + 0.011·X_1_·X_2_ − 0.022·X_1_·X_3_ + 0.054·X_1_·X_4_ − 0.002·X_2_^2^ + 4·10^−5^·X_2_·X_3_ + 0.018·X_2_·X_4_ − 0.496·X_3_^2^ + 0.005·X_3_ X_4_ + 0.019·X_4_^2^ (2)

The antioxidant capacity of the obtained extracts was further evaluated by the DPPH method. The results obtained were subsequently compared to the predicted values derived from the BBD-RSM. The average difference observed was 8.04%, ranging from 0.31% to 16.34%. Furthermore, the calculated R^2^ value was 0.75, indicating a satisfactory fit of the obtained results. The obtained Durbin-Watson value was 1.77, significantly higher than 0.05, indicating that the discrepancies between the calculated and predicted values were minimal.

After confirming the normal distribution of the results, an ANOVA was performed and the data were plotted in a Pareto chart (Figure 1B). Notably, the interaction between the %MeOH in the solvent and the extraction temperature emerged as the only significantly influential variable concerning antioxidant capacity. Similar to the observed in Figure 1A, this interaction demonstrated a positive effect, thus suggesting that higher values of both variables could increase the antioxidant capacity. Consequently, the optimal extraction conditions, considering only the maximization of the antioxidant capacity, were determined as follows: 0.5 g of sample extracted with 20 mL of solvent (composed of 74% MeOH at a pH of 2.7), with an extraction time of 15 min at 70 °C. In addition, the second-order Equation (3) was obtained to predict the antioxidant activity according to the developed BBD-RSM model:

Y = 114.106 + 0.982·X_1_ − 1.537·X_2_ − 3.752·X_3_ − 2.324·X_4_ − 0.054·X_1_^2^ + 0.059·X_1_·X_2_ − 0.116·X_1_·X_3_ − 0.028·X_1_·X_4_ − 0.004·X_2_^2^ + 0.004·X_2_·X_3_ + 0.022·X_2_·X_4_ + 0.263·X_3_^2^ + 0.038·X_3_·X_4_ + 0.014·X_4_^2^ (3)

After using the BBD-RSM design to identify the influential variables, the optimal extraction conditions to maximize both the total polyphenol concentration and the antioxidant capacity of the extracts were determined by a multi-parametric model (Figure S2). It was found that the polyphenol concentration and the antioxidant activity were enhanced as the %MeOH and the temperature increased. It was concluded that the ideal conditions include the use of 0.5 g of sample extracted with 20 mL of solvent consisting of 74% MeOH at pH 4.8 and 70 °C.

Regarding the solvent volume, it was found that the highest value within the evaluated range was optimal. However, considering the potential increase in energy consumption and cost associated with higher solvent volumes, and since this parameter had not previously been identified as an influential variable, it was considered unnecessary to explore higher values of this parameter. Similarly, although the optimal extraction temperature fell within the highest value of the temperature range, it is well-known that high temperatures can lead to polyphenol degradation [33]. Therefore, in order to preserve the integrity of the compounds, it was decided not to further increase the extraction temperature. Lastly, the percentage of MeOH was also found to be near the upper limit of the range. Nevertheless, it was decided to not explore higher values since the evaporation temperature of MeOH is ca. 65 °C. These considerations were made to ensure a balance between achieving optimal extraction conditions and mitigating potential drawbacks, such as excessive energy consumption, increased costs, and the risk of compound degradation or solvent evaporation.

### 3.2. Influence of Extraction Time

**Figure 2:**
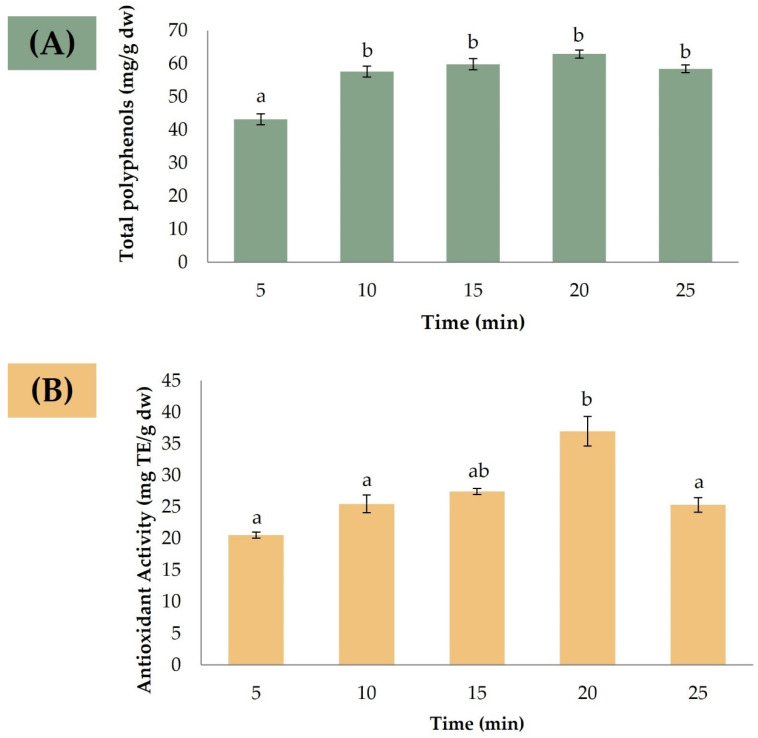
Effect of extraction time on the (**A**) total polyphenol concentration (mg/g dw) and (**B**) antioxidant activity (mg Trolox eq./g dw) of *A. unedo* pomace (*n* = 3). Different letters indicate significant differences at 95% confidence level.

Following the determination of optimal extraction conditions for *A. unedo* pomace, the study proceeded to evaluate the optimal extraction time. Extractions were performed in triplicate, using the established optimal conditions, at different times ranging from 5 to 25 min (i.e., 5, 10, 15, 20, and 25 min). The extracts were then analyzed to evaluate both the total polyphenol concentration and antioxidant activity (Figure 2A,B).

An ANOVA was used and revealed significant differences in both total polyphenol concentration and antioxidant activity among the various extraction times. A Duncan’s post-hoc analysis was conducted to further elucidate these differences. In terms of the total polyphenol concentration, it increases with increasing the extraction time (the peak concentration is reached at 20 min). Above 20 min, a decrease in total polyphenol concentration was observed, which could possibly be related to polyphenol degradation after prolonged exposure to high temperatures [34]. However, the ANOVA suggested no significant differences between the 10 and 25-min extraction times. Therefore, a 10-min extraction time was chosen to minimize energy consumption while still achieving a high total polyphenol concentration.

Regarding the antioxidant activity, a similar trend was observed, with an increase in activity up to 20 min extraction, followed by a decrease for longer extraction times. However, in this case, significant differences were observed except between the 15 and 20 min extraction times. Therefore, a 15-min extraction time was established as the optimal duration to achieve high antioxidant activity.

Overall, the data suggest that while longer extraction times may result in increased total polyphenol concentration and antioxidant activity, prolonged exposure may result in polyphenol degradation and reduced efficacy. Therefore, a balanced approach was taken, and 15 min was selected as the best compromise that maximized the desired results while mitigating the potential drawbacks associated with prolonged extractions.

### 3.3. Optimal Extraction Conditions

After the evaluation of the influential variables, three extractions were performed under the optimal conditions determined: 0.5 g of sample was extracted with 20 mL of solvent consisting of 74% MeOH at pH 4.8 and 70 °C for 15 min. The average concentration of total polyphenols achieved was 57.73 ± 2.05 mg/g dw, while the antioxidant activity reached 30.00 ± 0.912 mg TE/g dw.

To the best of the author’s knowledge, there is only one study that has investigated the extraction of anthocyanins from *A. unedo* pomace [14], but using a supercritical fluid extraction approach. The most promising conditions obtained in this study involved extraction with 20% ethanol in CO_2_ at 55 °C and 300 bar. However, the use of such pressurized systems significantly increases the energetic cost compared to the USE conditions identified in the current study.

Other researchers have investigated the use of USE for extracting anthocyanins from fresh *A. unedo* fruits. López et al. [35] found that the optimal conditions to achieve the highest concentration of anthocyanins were 1.5 g of sample with 50 mL of solvent (60% EtOH) at 243.7 W for 27.6 min at 35 °C, resulting in a total anthocyanin concentration of 0.80 ± 0.036 mg/g dw. While the solvent percentages and ratios were similar to those used in the current study, longer extraction times are required. Note that the concentrations achieved for total anthocyanins were notably higher in the pure fruit extracts due to the absence of a pre-extraction step during the distillation process.

Albuquerque et al. [26] investigated the extraction of catechins from *A. unedo* fruits using USE, employing the following conditions: 2.5 g of sample with 50 mL of solvent (40% MeOH) for 45 min at 315 W, resulting in a catechin concentration of 0.71 ± 0.1 mg/g dw. In this case, longer extraction times and larger solvent volumes were utilized which potentially makes the process more costly. Subsequently, the authors [36] examined the USE extraction of polyphenols from fresh *A. unedo* fruits, setting the optimal conditions as 2.5 g of sample extracted with 50 mL of solvent for 120 min at 90 W, using 60% MeOH at 35 °C. These conditions yielded a total polyphenol concentration of 646.1 ± 91.6 mg/g dw and an antioxidant activity of 0.658 ± 0.004 nM DPPH/μg dw.

Lastly, El Cadi et al. [37] assessed the total polyphenol concentrations in *A. unedo* fruits using the following conditions: 5 g of sample defatted with 50 mL of n-hexane (repeated three times), dried, and homogenized with 50 mL of two solvents with increasing polarity (EtOAc or MeOH:H_2_O, 80:20 *v*/*v*). Each fraction was then extracted via sonication in an ultrasonic bath (130 kHz) for 45 min. It was observed that the MeOH:H_2_O (4:1) resulted in a higher extraction yield of 75.88 ± 3.1 mg/g dw compared to the EtOAc extraction (51.61 ± 0.98 mg/g dw). This agrees well with the optimal percentage of MeOH identified in the current study. However, the concentrations obtained in that study were significantly lower than those expected from the extraction of the pure fruit. There is strong evidence that the fermentation and/or distillation processes may significantly affect the total polyphenol content. In fact, Rodrigues et al. [15] observed that the total flavonoid content of *A. unedo* pomace was two times higher than that of the fruit.

As it was previously mentioned, the extracts obtained under optimal conditions were analyzed by UHPLC-PDA-Q-Tof-MS-QDa, and three main polyphenols were identified: gallic acid, 5-*O*-galloylquinic acid, and ethyl gallate. Polyphenols that have been extensively identified in *A. unedo* fruits [36,37], confirming their presence in the pomace.

### 3.4. Validation of the Extraction Method

Upon completion of the optimization process, the developed extraction method was subjected to a validation study to assess the repeatability and intermediate precision. The validation study consisted of performing nine experiments on three different days, resulting in a total of 27 experiments under the determined optimal conditions.

Repeatability was evaluated by performing nine experiments on the same day, while the intermediate precision was assessed by collecting data from the experiments performed on three different days (nine experiments per day).

For the repeatability, the total polyphenol concentration achieved was 57.73 ± 2.05 mg/g dw, while the antioxidant activity reached 30.00 ± 0.91 mg TE/g dw. For the intermediate precision, the total polyphenol concentration achieved was 57.07 ± 1.69 mg/g dw, while the antioxidant activity was 28.81 ± 1.01 mg TE/g dw. The coefficient of variation (C.V.) was used as a statistical measure for the validation study. The C.V. values for repeatability were 3.56% and 3.06% for total polyphenols and antioxidant activity, respectively. On the other hand, the C.V. values for intermediate precision were 2.96% and 3.51% for total polyphenols and antioxidant activity, respectively. As observed, all C.V. values were below 5%, thus indicating that the developed method is reliable, repeatable, and presents good intermediate precision.

### 3.5. Development of Functional Cookies with A. unedo Pomace

#### 3.5.1. Sensory Evaluation

In the first part of this study, the high content of polyphenols in *A. unedo* pomace and their associated antioxidant activity was demonstrated. In addition, the use of *A. unedo* pomace as a raw material in the development of a proof-of-concept functional food was evaluated. For that, homemade cookies were prepared by partially replacing the standard flour with different fractions of freeze-dried pomace (0% to 60%) (see Section 2.4. for details). Once the cookies were baked, they were subjected to a sensory evaluation by a panel of experts composed of 52 individuals from Spain and 15 individuals from Portugal. Among the panelists, 48% were male and 52% were female. The age of the participants ranged from 18 to 65 years, with the largest group (approximately 30%) being between 26 and 35 years old. The results of this survey are presented in Table S1.

The panelists were given an evaluation questionnaire that consisted of two tests. The first test was designed to assess their ability to discriminate between cookies. They were presented with three cookies, two of which contained 0% pomace, and one containing 20% pomace powder. The panelists were asked to discriminate the pomace-containing cookies based on visual appearance, aroma, texture, and taste. The results revealed that 84% of the panelists were able to visually distinguish the pomace-containing cookies, while 73% and 75% were able to do so based on aroma and texture, respectively. In terms of taste discrimination, 67% of the panelists were able to differentiate the cookies containing pomace.

The second test involved the evaluation of five cookies with varying amounts of pomace, ranging from 0% to 40%. Again, the panelists were asked several questions pertaining to their perception of the cookies in terms of appearance, aroma, texture, and taste. Most participants found all cookies to be palatable, even those with 40% pomace, except in the case of texture, where the majority considered unpalatable the cookie with the highest fraction of pomace.

When the participants were asked specific questions regarding their preferences, it was observed that 22% found the taste of the cookie with 15% pomace pleasant, while 18% found the taste of the cookie with 20% pomace pleasant. Additionally, 35% of the participants expressed their willingness to purchase the cookie with 10% pomace, while 28% and 26% showed interest in the cookies with 15% and 20% pomace, respectively. Participants expressed dissatisfaction with the hardness of the seeds present in the mixture. Therefore, it was decided to produce cookies with *A. unedo* pomace, after removing the seeds using a commercial sieve with a pore size of 0.22 mm.

The cookie preparation process was essentially the same as previously described. However, to better address the texture issue, the percentage of pomace was increased up to 60%. Note that higher percentages were not evaluated since the consistency of the dough was compromised. To evaluate the seedless formulations, 20% of the panelists who had previously evaluated cookies with seeds were retained (randomly selected), while the remaining 60% were new panelists. This included 40 panelists from Spain and 8 panelists from Portugal. The gender distribution was 42% male and 58% female. The age range was between 18 and 65 years, with the majority falling within the 26 to 35 years age group. The results of these surveys are shown in Table S2.

In this case, the evaluation focused on assessing the tasters’ preferences for different percentages of *A. unedo* pomace, ranging from 0% to 60%. Most of the panelists did not find any of the cookies unpalatable in terms of appearance, texture, or smell. However, most tasters found the cookie containing 60% pomace unpalatable in terms of taste. Remarkably, the appreciation for the cookie containing 40% pomace residue increased significantly compared to the initial survey.

**Figure 3:**
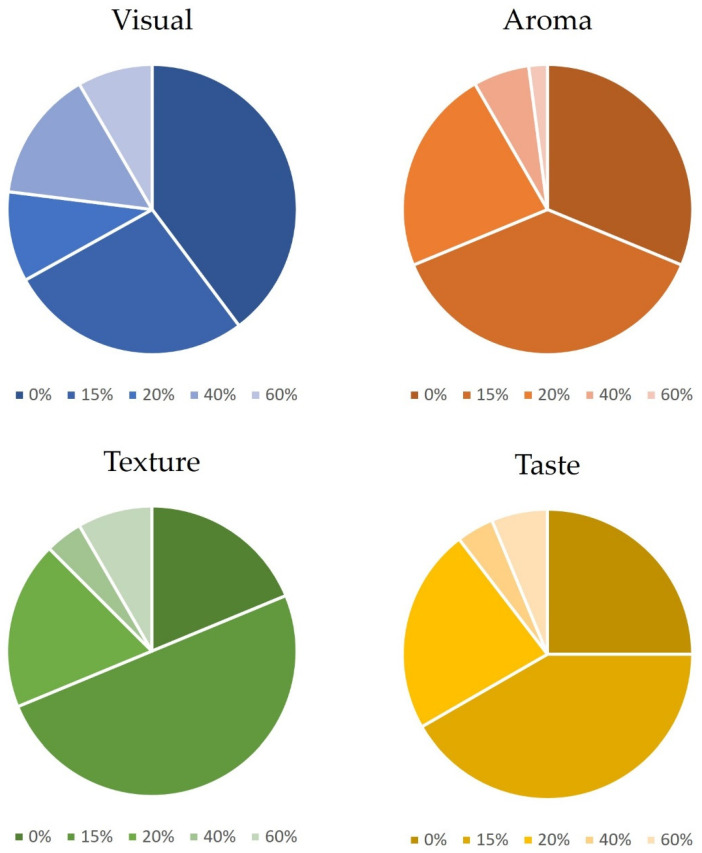
Summary of the panelists’ evaluation of the cookies developed with the seedless *A. unedo* pomace powder (i.e., 0%, 15%, 20%, 40%, and 60%).

A statistical analysis was conducted to evaluate the panelists’ preferences for cookies based on visual appearance, aroma, texture, and taste. The results are depicted in Figure 3.

As can be seen, most panelists rated the cookies with 15% pomace better (in terms of aroma, texture and flavor) than those backed with the original formulation without pomace. Regarding the cookies with 20% pomace, their appreciation was nearly identical to those containing 0% residue in terms of texture and flavor, but lower in terms of aroma and visual appeal. Therefore, it was concluded that this threshold (i.e., 20%) represents the maximum acceptable level of pomace in the formulation while still being appreciated by consumers.

Tasters were also asked to provide simple descriptions of the cookies with the highest percentage of pomace (i.e., 60%) in terms of visual appearance, aroma, texture, and taste. Responses revealed that 35% described the cookie as dark or burnt, 31% found it pleasant, 15% perceived it as oily, and 13% visually associated it with caramelized or chocolate. In terms of aroma, common remarks included describing it as vegetable and strong (23%), as fruity, sweet, and caramelized (17%), and as oily (4%). Regarding the texture, the majority (57%) described it as soft and non-crisp, while 24% found the texture suitable for a cookie and 20% characterized it as sandy. In terms of flavor, 83% associated it with acidity and to a strong flavor, while only 17% associated it with sweetness and caramelized flavors. Interestingly, significant differences were noted in the evaluation of flavor depending on the region studied. In the Portuguese region, where this fruit is commonly consumed, markers such as sweetness, ripe fruit, caramel, or chocolate were used to describe the cookie. In contrast, in the Spanish region, markers were more related to acidity, strength, or astringency.

Finally, 85% of the participants expressed their intention to purchase cookies containing 15% pomace, while 65% indicated a willingness to purchase the cookies containing 20% pomace. Only 24% expressed their interest in purchasing cookies with 40% pomace while the lowest willingness (13% of the panelists) was observed for the cookies with 60% pomace residue.

#### 3.5.2. Polyphenolic Content and Antioxidant Activity of the Cookies

After the detailed sensory analysis discussed in the previous section, the cookies were also chemically analyzed. To perform such analysis, the cookies were grounded and then the polyphenols were extracted using the optimized USE method developed for the *A. unedo* pomace (see Section 3.3 for details).

**Figure 4:**
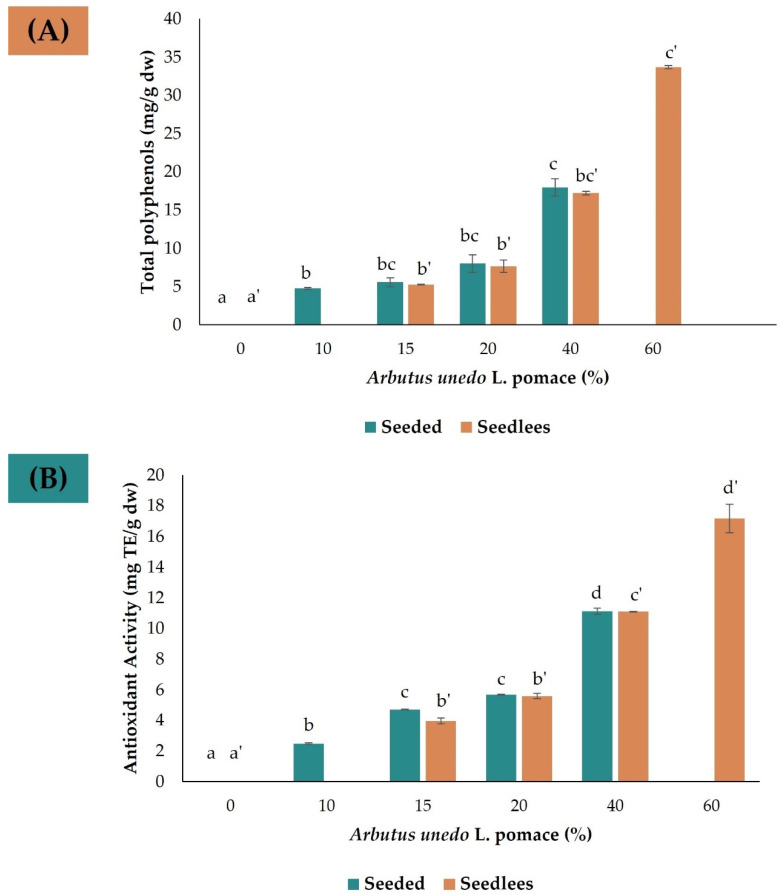
(**A**) Total polyphenol concentration (mg/d dw) and (**B**) antioxidant activity (mg TE/g dw) of the extracts obtained from cookies with and without seeds analyzed under optimal extraction conditions. Different letters indicate significant differences at 95% confidence level.

As anticipated, the first observation was that the concentration of polyphenols present in the cookies increased with the amount of pomace used in the formulation (Figure 4). The total polyphenol concentration ranged from an average of 4.76 mg/g dw (10% pomace) to 17.97 mg/g dw (40% pomace). Comparing these concentrations with the theoretically expected values based on the analysis carried out in the native pomace, a decrease in polyphenol concentration is noticed. Such decrease is, most likely, due to both the volatile profile of some polyphenols and possible degradation during the backing process. Remarkably, despite the harsh backing conditions (i.e., 180 °C for 10–12 min) the polyphenol decrease was less than 26% in all cases.

As previously discussed, the extracts obtained from cookies were analyzed by UHPLC-PDA-Q-Tof-MS-QDa, and the area of the three main polyphenols identified (i.e., gallic acid, 5-*O*-galloylquinic acid, and ethyl gallate) was calculated and compared with the theoretical value based on the extracts obtained under optimal conditions. It is observed that the biggest change occurs to the gallic acid with a difference between the theorical concentration and the observed of 25.85%, followed by galloylquinic acid (25.39%), and ethyl gallate (16.67%).

The antioxidant activity of the extracts obtained from the baked cookies follows the same trend and is also observed to increase with the amount of pomace incorporated in the cookies. Samples were also compared regarding the effect of the seeds in the cookie’s formulation. Overall, no significant differences (<5%) were found between the total polyphenol content and antioxidant capacity of seeded and seedless cookies, suggesting that fruit polyphenols are mainly distributed between the skin and pulp.

As mentioned above, the seedless formulations demonstrated that the optimal amount of pomace for consumer acceptance fell within the range of 15% to 20%. This would result in an intake of ca. 7.90 mg of polyphenols per gram of consumed cookie, accompanied by an antioxidant activity of ca. 5.67 mg Trolox eq. per gram of consumed cookie. Such a contribution is of great importance in daily polyphenol intake, as it may significantly contribute to prevent cardiovascular diseases, diabetes, and even cancer.

A comprehensive comparison with the existing literature poses a significant challenge due to the predominant focus on the interaction between food matrices, particularly meat or milk [38,39,40], and polyphenols-enriched extracts derived from plants or other food sources. The primary emphasis has been on enhancing the properties of these matrices, such as antioxidant activity and shelf life, rather than exploring the final composition of the matrices or the development of functional foods. Nevertheless, there have been noteworthy investigations in this area. For example, Törrönen et al. [41] conducted a study involving the incorporation of crowberry powder, rich in polyphenols, into blackcurrant juice, resulting in a final polyphenolic composition of 2.93 mg/mL. Similarly, Mildner-Szkudlarz et al. [42] developed cookies by incorporating white grape pomace, with the most favorable consumer response observed at a 10% concentration, resulting in a final polyphenolic content of 0.30 mg per gram and an antioxidant activity of 7.55 mmol Trolox equivalents per gram of cookie. Wang et al. [43] investigated the incorporation of green tea extracts in the production of bread and found that the addition of 0.15 mg of green tea per gram of flour resulted in a detectable level of 0.52 mg of catechins per gram of bread. A related study, Bajerska et al. [44] investigated the incorporation of green tea into bread preparation and found that the most preferred concentration by consumers was 0.8%. At this level, the bread exhibited a total polyphenol concentration of 3943.9 mg/kg and an antioxidant activity of 13.2 mmol Trolox equivalents per gram of bread. In another study, López-López et al. [45] evaluated the use of sea spaghetti in the formulation of frankfurters, resulting in a concentration of 25.7 mg of polyphenols/g of the frankfurters. This incorporation resulted in a significant increase in polyphenol concentration. Similarly, Ayo et al. [46] achieved a concentration of 5.6 mg/g in frankfurters supplemented with 25% walnuts.

The findings from the present study demonstrate significant progress in the development of functional foods with high polyphenolic content, which can be incorporated into diets to promote health and provide considerable antioxidant benefits.

## 4. Conclusions

The present study focused on the optimization of ultrasound-assisted extraction of polyphenols from *A. unedo* fruit distillate residues (pomace). A Box-Behnken design-response surface methodology was conducted, revealing that the percentage of methanol (solvent used for extraction), the temperature, and their interactions were the most influential variables affecting the extraction of total polyphenols and antioxidant activity of the extracts. In addition, extraction time was found to have a significant effect on both parameters. The optimized conditions determined to maximize polyphenol content and antioxidant activity were as follows: 0.5 g of sample extracted with 20 mL of a solvent composed of 74% MeOH (aq), with pH 4.8, maintained at 70 °C for 15 min. In addition, the developed extraction method demonstrated excellent repeatability and intermediate precision, with C.V. values lower than 5%.

The *A. unedo* pomace was further evaluated regarding its direct incorporation in the development of novel functional cookies. Notably, the cookies containing 15% to 20% of pomace were highly appreciated, even surpassing in various parameters the original cookie formulation with 0% pomace. This translates to a significant contribution of ca. 7.90 mg of polyphenols per gram of consumed cookie, accompanied by an antioxidant activity of ca. 5.67 mg Trolox equivalent per gram of consumed cookie. It should be highlighted that such concentrations are significantly higher than those observed in related functional foods reported in the literature.

To the best of our knowledge, this was the first time an USE-based optimized method has been developed to maximize the extraction of phenolic compounds from *A. unedo* pomace. Moreover, this work also showcases the applicability of the pomace in daily consumption, via the development of novel highly appealing functional cookies. The consumption of these cookies could substantially increase the intake of bioactive compounds in our daily diet, potentially offering significant health benefits. Additionally, by valorising a sub-product currently discarded and generated in substantial quantities, particularly in the Algarve region, this work supports the reduction of environmental pollution while valorising agroindustry residues potentially generating novel business opportunities while contributing to circular economy.

## Figures and Tables

**Table 1:** BBD-RSM design for total polyphenol content and antioxidant activity of *A. unedo* pomace extracts.

					Total Polyphenols (mg/g dw)			Antioxidant Activity (mg TE/g dw)		
Experiment	Ratio (0.5 g/mL Solvent)	%MeOH	pH	Temperature (°C)	Observed	Adjusted	Error (%)	Observed	Adjusted	Error (%)
1	20	25	4.5	50	35.41 ± 2.56	31.92	9.85	10.09 ± 0.37	9.48	6.05
2	15	75	4.5	30	35.93 ± 1.45	35.48	1.24	17.19 ± 6.70	16.76	2.50
3	15	75	2	50	35.30 ± 6.12	36.75	4.10	20.62 ± 5.26	20.17	2.15
4	20	50	4.5	30	34.94 ± 2.20	36.18	3.55	25.35 ± 3.35	27.63	8.98
5	20	50	7	50	33.18 ± 3.03	34.38	3.60	16.67 ± 1.49	15.80	5.20
6	15	25	4.5	70	42.72 ± 0.93	36.84	13.78	6.04 ± 2.47	6.64	9.90
7	20	50	4.5	70	46.25 ± 6.43	53.59	15.87	18.04 ± 8.19	19.87	10.16
8	10	25	4.5	50	24.66 ± 1.12	25.23	2.33	17.41 ± 2.23	17.32	0.49
9	15	50	2	70	37.70 ± 2.27	36.08	4.31	20.88 ± 9.68	24.29	16.34
10	15	50	7	70	48.65 ± 0.42	47.63	2.09	26.14 ± 4.47	23.06	11.78
11	10	50	7	50	30.92 ± 0.96	29.34	5.11	15.64 ± 2.20	14.68	6.18
12	10	75	4.5	50	24.97 ± 0.97	21.49	13.94	5.28 ± 2.23	5.48	3.70
13	15	50	2	30	36.07 ± 0.45	40.12	11.24	22.98 ± 3.72	20.20	12.10
14	10	50	4.5	30	45.52 ± 4.73	41.48	8.87	25.09 ± 0.74	27.94	11.38
15	15	25	2	50	33.19 ± 1.22	31.59	4.81	24.04 ± 3.72	27.68	15.15
16	15	50	4.5	50	44.47 ± 4.73	39.07	12.13	18.93 ± 3.21	17.46	7.79
17	15	50	4.5	50	37.53 ± 0.95	39.07	4.10	17.09 ± 3.70	17.46	2.14
18	15	75	4.5	70	71.33 ± 5.52	60.46	15.24	37.19 ± 7.09	32.03	13.89
19	20	50	2	50	38.69 ± 3.15	33.94	12.27	25.61 ± 6.70	23.74	7.30
20	10	50	2	50	35.32 ± 4.02	37.79	6.99	18.77 ± 1.49	16.81	10.46
21	15	25	7	50	30.72 ± 0.06	32.58	6.05	12.98 ± 0.00	12.11	6.74
22	15	50	7	30	45.90 ± 1.70	40.55	11.64	20.62 ± 5.30	21.36	3.59
23	15	75	7	50	32.84 ± 1.03	37.74	14.91	15.62 ± 4.09	15.67	0.31
24	10	50	4.5	70	35.04 ± 3.37	37.10	5.88	25.09 ± 0.74	21.50	14.32
25	15	50	4.5	50	35.22 ± 4.01	39.07	10.93	15.35 ± 1.33	17.46	13.71
26	20	75	4.5	50	41.53 ± 2.76	39.99	3.71	26.14 ± 0.74	24.38	6.75
27	15	25	4.5	30	44.26 ± 2.92	48.79	10.25	28.77 ± 1.49	31.10	8.11

## Data Availability

Data will be available under request.

## Supplementary Materials

The following supporting information can be downloaded at: https://www.mdpi.com/article/10.3390/foods12193707/s1, Figure S1. (A) QR code to Google Form survey employed by the panelist to evaluate the cookies; (B) Testing rooms from Penha campus, University of Algarve; and (C) Testing rooms from IFAPA Rancho La Merced. Figure S2. Response Surface Graph from multi-parametric analysis considering the total polyphenol concentration and antioxidant activity. The desirability (maximum polyphenol content and antioxidant activity of the extracts) was normalized. Note that the red color was assigned to the highest desirability (1), while the blue color was assigned to the lowest desirability (0). Table S1. Survey answers obtained from the sensory evaluation of cookies with seeds. Table S2. Survey answers obtained from the sensory evaluation of cookies without seeds.
