# Supplementary material for: On the Valorization of Arbutus unedo L. Pomace: Polyphenol Extraction and Development of Novel Functional Cookies
Source: Foods. 2023 Oct 9;12(19):3707. doi: 10.3390/foods12193707 (PMC10572809; doi:10.3390/foods12193707)
Supplement: Supplementary file 1 [file foods-12-03707-s001.zip › Table S1.pdf]

foods

Table S1. Survey answers obtained from the sensory evaluation of cookies with seeds.

**Parameter**	*n*	%
	**Country**	
Spain	52	78
Portugal	15	22
	**Gender**	
Masculine	32	48
Feminine	35	52
Non-binary	0	0
	**Age**	
18-25	8	12
26-35	20	30
36-45	8	12
46-55	15	22
56-65	15	22
>65	1	1
	**Test 1. 1 and 2: 0%; 3: 20%**	
	1. Visually, do you find any cookies different from the rest?	
1	4	6
2	3	4
3	56	84
None	0	0
All	4	6
	2. Do you find any cookies different from the rest because of their smell?	
1	7	10
2	3	4
3	49	73
None	7	10
All	1	1
	3. Do you find any cookies different from the rest because of their flavor?	
1	6	9
2	6	9
3	45	67
None	7	10
All	3	4
	4. Do you find any cookies different in texture in hand or mouth?	
1	5	7
2	3	4
3	50	75
None	9	13
All	0	0

**Test 2 (multiresponse). 1: 0%; 2: 10%; 3: 20%; 4: 15%; 5: 40%**		
1. Visually, are there any cookie(s) that you find objectionable?		
1	0	0
2	6	8
3	6	8
4	5	6
5	17	22
None	43	56
All	0	0
2. Do you find the smell of any or some cookies unpleasant?		
1	2	3
2	1	1
3	5	7
4	4	5
5	25	33
None	39	51
All	0	0
3. Do you find the texture in hand and/or mouth of any or some cookies unpleasant?		
1	1	1
2	8	9
3	17	18
4	15	16
5	31	33
None	22	23
All	0	0
4. Is the taste of any or some cookies unpleasant to you?		
1	0	0
2	3	4
3	6	7
4	8	10
5	25	31
None	39	48
All	0	0
5. On a visual level, which cookie do you find most pleasing?		
1	18	27
2	17	25
3	12	18
4	13	19
5	7	10
6. On a visual level, which cookie do you find most unpleasing?		
1	9	13
2	8	12

3	10	15
4	9	13
5	31	46
7. On an olfactory level, which cookie do you find most pleasant?		
1	17	25
2	13	19
3	11	16
4	17	25
5	9	13
8. On an olfactory level, which cookie do you find most unpleasant?		
1	6	9
2	3	5
3	4	6
4	4	6
5	47	73
9. In terms of texture, which cookie do you find more pleasant?		
1	19	28
2	18	27
3	12	18
4	15	22
5	3	4
10. In terms of texture, which cookie do you find more unpleasant?		
1	1	1
2	0	0
3	16	24
4	4	6
5	46	69
11. In terms of taste, which cookie do you find most pleasant?		
1	20	30
2	12	18
3	12	18
4	15	22
5	8	12
12. In terms of taste, which cookie do you find most unpleasant?		
1	9	13
2	2	3
3	7	10
4	4	6
5	45	67
13. Would you buy any cookies from 2-5? Select all that you would buy		
2	44	35
3	33	26

4	36	28
5	9	7
None	1	1
All	4	3
