# Supplementary material for: On the Valorization of Arbutus unedo L. Pomace: Polyphenol Extraction and Development of Novel Functional Cookies
Source: Foods. 2023 Oct 9;12(19):3707. doi: 10.3390/foods12193707 (PMC10572809; doi:10.3390/foods12193707)
Supplement: Supplementary file 1 [file foods-12-03707-s001.zip › Table S2.pdf]

foods

MDPI

**Table S2**. Survey answers obtained from the sensory evaluation of cookies without seeds.

| Parameter | *n* | % |
|---|---|---|
| | **Country** | |
| Spain | 40 | 83 |
| Portugal | 8 | 17 |
| | **Gender** | |
| Masculine | 20 | 42 |
| Feminine | 28 | 58 |
| Non-binary | 0 | 0 |
| | **Age** | |
| 18-25 | 5 | 10 |
| 26-35 | 13 | 27 |
| 36-45 | 6 | 13 |
| 46-55 | 9 | 19 |
| 56-65 | 8 | 17 |
| >65 | 1 | 2 |
| | **(1):0%; (2):20%; (3):15%; (4):40%; (5):60%** | |
| | *1. Visually, are there any cookie(s) that you find unpleasant?* | |
| None | 28 | 49 |
| 1 | 6 | 11 |
| 2 | 5 | 9 |
| 3 | 6 | 11 |
| 4 | 3 | 5 |
| 5 | 9 | 16 |
| | *2. On a visual level, which cookie do you find most unpleasant?* | |
| 1 | 7 | 15 |
| 2 | 6 | 13 |
| 3 | 7 | 15 |
| 4 | 4 | 8 |
| 5 | 24 | 50 |
| | *3. On a visual level, which cookie do you find most pleasant?* | |
| 1 | 19 | 40 |
| 2 | 5 | 10 |
| 3 | 13 | 27 |
| 4 | 7 | 15 |
| 5 | 4 | 8 |
| | *4. Do you find the smell of any cookie(s) unpleasant?* | |
| None | 23 | 38 |
| 1 | 1 | 2 |
| 2 | 3 | 5 |
| 3 | 1 | 2 |

| | | |
|---|---|---|
| 4 | 13 | 22 |
| 5 | 19 | 32 |
| *5. Which cookie do you find most unpleasant by its smell?* | | |
| 1 | 3 | 6 |
| 2 | 1 | 2 |
| 3 | 2 | 4 |
| 4 | 9 | 19 |
| 5 | 33 | 69 |
| *6. Which cookie do you find most pleasant by its smell?* | | |
| 1 | 15 | 31 |
| 2 | 11 | 23 |
| 3 | 18 | 38 |
| 4 | 3 | 6 |
| 5 | 1 | 2 |
| *7. Do you find the texture in your hand and/or mouth of any or some cookies unpleasant?* | | |
| None | 24 | 41 |
| 1 | 4 | 7 |
| 2 | 5 | 8 |
| 3 | 3 | 5 |
| 4 | 6 | 10 |
| 5 | 17 | 29 |
| *8. Texturally, which cookie do you find the most unpleasant?* | | |
| 1 | 4 | 8 |
| 2 | 1 | 2 |
| 3 | 1 | 2 |
| 4 | 9 | 19 |
| 5 | 33 | 69 |
| *9. Texturally, which cookie do you find the most pleasant?* | | |
| 1 | 9 | 19 |
| 2 | 9 | 19 |
| 3 | 24 | 50 |
| 4 | 2 | 4 |
| 5 | 4 | 8 |
| *10. Is the taste of any or some cookies unpleasant to you?* | | |
| None | 17 | 27 |
| 1 | 2 | 3 |
| 2 | 2 | 3 |
| 3 | 0 | 0 |
| 4 | 16 | 25 |
| 5 | 27 | 42 |
| *11. On a taste level, which cookie do you find most unpleasant?* | | |
| 1 | 1 | 2 |

| | | |
|---|---|---|
| 2 | 2 | 3 |
| 3 | 2 | 3 |
| 4 | 5 | 8 |
| 5 | 38 | 59 |
| *12. On a taste level, which cookie do you find most pleasant?* | | |
| 1 | 12 | 25 |
| 2 | 11 | 23 |
| 3 | 20 | 42 |
| 4 | 2 | 4 |
| 5 | 3 | 6 |
| *13. Would you buy any cookies from 2-5? Select all that you would buy* | | |
| 2 | 31 | 65 |
| 3 | 41 | 85 |
| 4 | 11 | 23 |
| 5 | 6 | 13 |
| None | 0 | 0 |
| All | 0 | 0 |
