# Supplementary figures and images for: On the Valorization of Arbutus unedo L. Pomace: Polyphenol Extraction and Development of Novel Functional Cookies
Source: Foods. 2023 Oct 9;12(19):3707. doi: 10.3390/foods12193707 (PMC10572809; doi:10.3390/foods12193707)

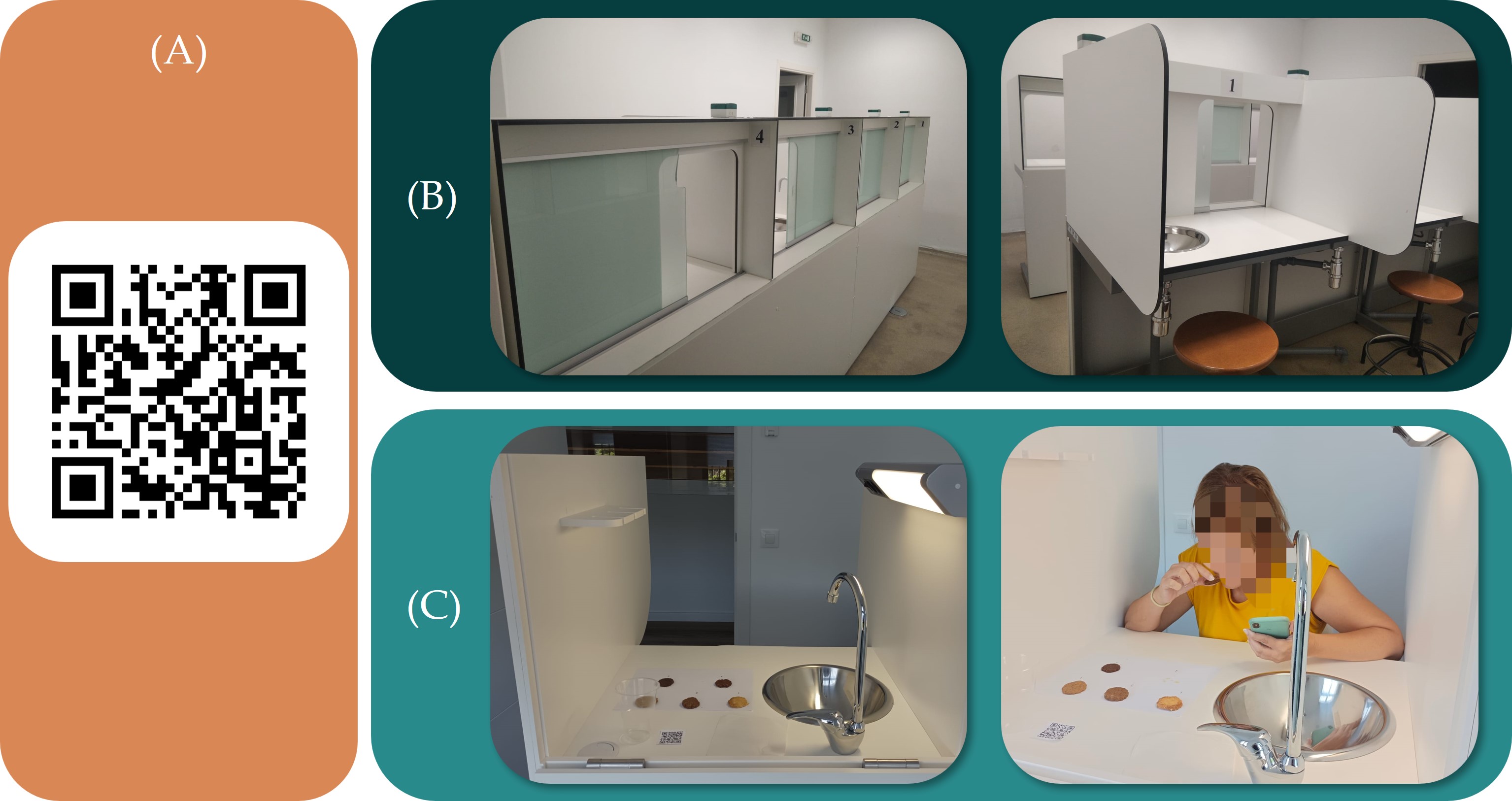

Supplement: Supplementary file 1 [file foods-12-03707-s001.zip › Figure S1.jpg]

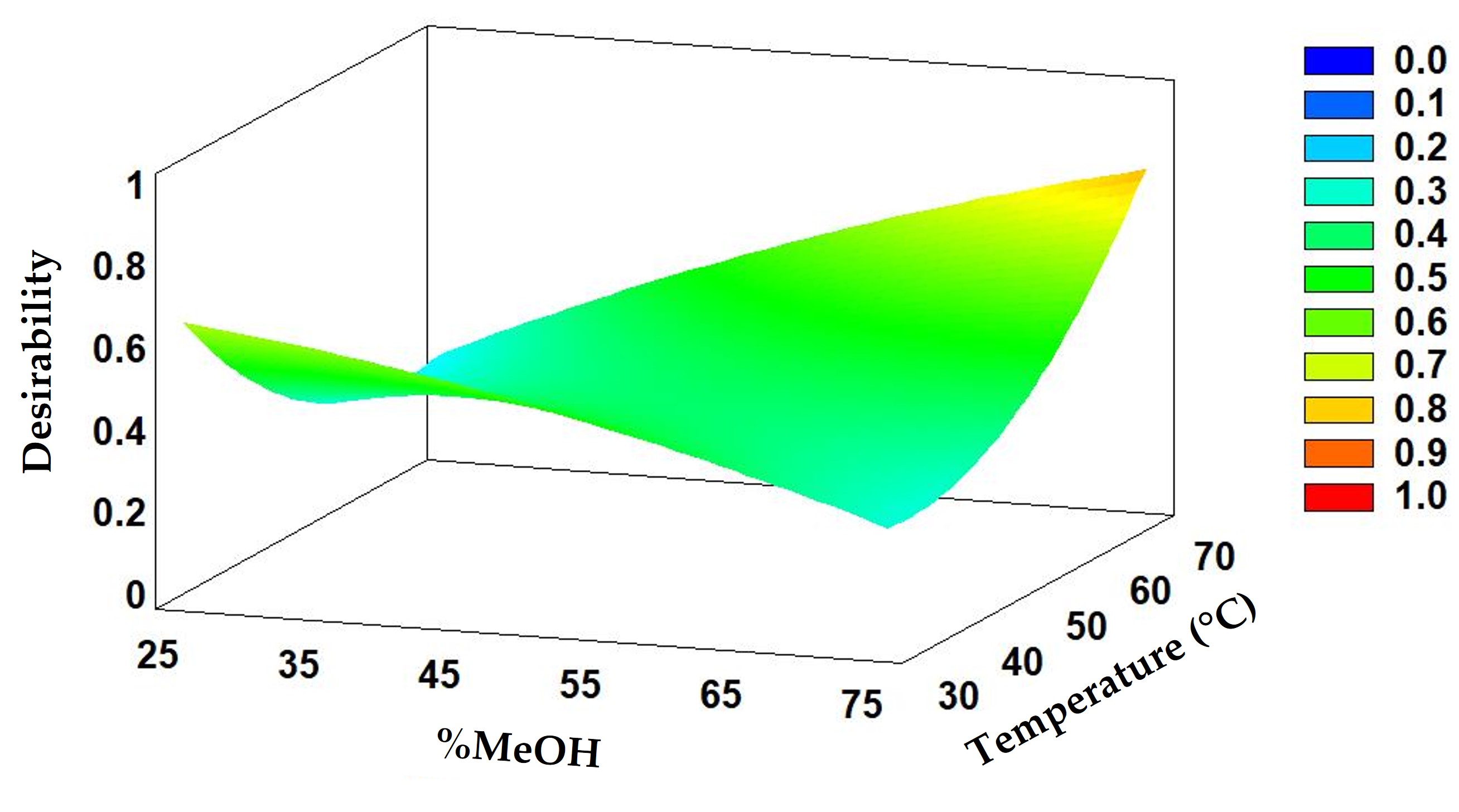

Supplement: Supplementary file 1 [file foods-12-03707-s001.zip › Figure S2.jpg]
